# A spider in motion: facets of sensory guidance

**DOI:** 10.1007/s00359-020-01449-z

**Published:** 2020-11-02

**Authors:** Friedrich G. Barth

**Affiliations:** Department of Neurosciences and Developmental Biology, Faculty of Life Sciences, University of Vienna, Althanstr.14, 1090 Vienna, Austria

**Keywords:** Spider motion, Sensory control, Mechanoreception, Sensory ecology, Neuroethology

## Abstract

Spiders show a broad range of motions in addition to walking and running with their eight coordinated legs taking them towards their resources and away from danger. The usefulness of all these motions depends on the ability to control and adjust them to changing environmental conditions. A remarkable wealth of sensory receptors guarantees the necessary guidance. Many facets of such guidance have emerged from neuroethological research on the wandering spider *Cupiennius salei* and its allies, although sensori-motor control was not the main focus of this work. The present review may serve as a springboard for future studies aiming towards a more complete understanding of the spider’s control of its different types of motion. Among the topics shortly addressed are the involvement of lyriform slit sensilla in path integration, muscle reflexes in the walking legs, the monitoring of joint movement, the neuromuscular control of body raising, the generation of vibratory courtship signals, the sensory guidance of the jump to flying prey and the triggering of spiderling dispersal behavior. Finally, the interaction of sensors on different legs in oriented turning behavior and that of the sensory systems for substrate vibration and medium flow are addressed.

## Introduction

*Neuroethology* aims at an understanding of the neural and sensory mechanisms underlying behavior, which most obviously manifests itself as motion. Motion, in turn, is primarily thought of as locomotion taking the animal from one place to another, as rhythmic motion like walking, running and flying. However, the motion also implies a large range of other ethologically relevant movements associated with prey capture, courtship, idiothetic orientation, and dispersal behavior, to name just a few examples.

A lot still has to be learned regarding the sensory feedback control of motive behavior in spiders and the relevant proximal sensory cues, which allow the necessary ad hoc adaptations to the environment. So far, the study of spider motion has focused on questions of physics and engineering, such as how muscles, the hydraulic leg extension system and the exoskeletons generate and support the forces enabling locomotion. Much less attention has been given to its sensory control. This is also seen when looking at the list of contents of the present Special Issue, which, therefore, provides a welcome platform to draw attention to this deficit.

Several lines of our neuroethological research on the Central American wandering spider *Cupiennius salei* and its close relatives, although often with a different focus, have revealed a number of interesting aspects of the sensory guidance of a variety of motions. The present review reports some of these findings. It is not a novelty paper but shortly summarizes what might serve as a springboard for future research. The field is still wide open and there are numerous problems deserving renewed interest by neuroethology and sensory biology, not to mention the open questions regarding kinematics (analysis of movement) and dynamics (analysis of forces and torques).

### *Cupiennius*—a model spider

**Fig. 1:**
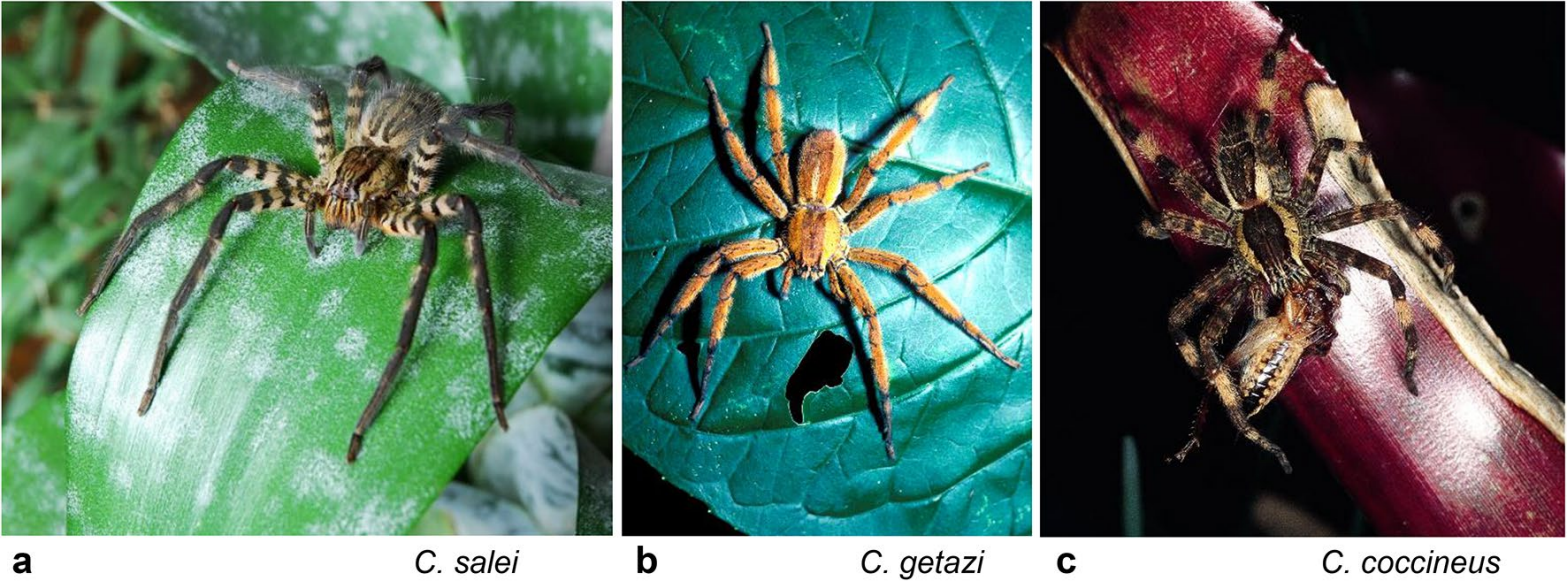
The three large *Cupiennius* species which turned out be perfect “model spiders “. **a** *Cupiennius salei*, **b** *C. getazi*, **c** *C. coccineus* (fotos FG Barth; with permission of SpringerNature; modified from **a** Barth 2015a; **c** Barth 2002b.)

*Cupiennius salei* and its closest relatives, *C. coccineus* and *C. getazi*, are a group of similarly large and robust neotropical spiders with leg spans of 10 cm and more. Considering their prominent role in the research described below, their outstanding importance gained in many other fields of research (Barth 2002a, b, 2008; McGregor et al. 2008), and their usefulness for future research these spiders shall shortly be introduced here (Fig. 1a–c).

The family assignment of the genus *Cupiennius* has been with the Ctenidae (Lachmuth et al. 1984; Barth 2002a, b; Barth and Cordes 2008) since Simon (1891) first placed them there almost 130 years ago. Recently, however, based mainly on molecular phylogeny, the genus was moved into the “fishing spiders “, Trechaleidae (Piacentini and Ramirez 2019), a sister group of the wolf spiders (Lycosidae).

In the context of the present Special Issue of JCP-A and future research on sensory-motor integration a few practical aspects may be more relevant to communicate than knowledge of the taxonomic assignment. The three large species of *Cupiennius* (*C. salei, C. getazi, C. coccineus*) can easily be bred in large numbers, thus being readily available for a large range of laboratory work. They are wandering spiders, not using a web for prey capture, which makes their handling and study much easier than that of delicate spiders living in fragile webs. The three big *Cupiennius* species are robust spiders well-tolerating handling and many experimental procedures. All of these qualities have contributed to make *Cupiennius* a model spider much appreciated in many laboratories worldwide for a broad spectrum of research. Existing studies include research in general biology, sensory physiology, neuroanatomy, biomechanics, exoskeletal structure and functional material properties, venom and hemolymph biochemistry, circulatory system, muscular metabolism, ecology, evolutionary developmental biology, taxonomy, friction control, and more (reviews: Barth 2002a, b, 2008; McGregor et al. 2008; some examples: Melchers 1963; Seitz 1966; Linzen et al. 1985; Paul et al. 1994; Fabian-Fine et al. 2000, 2002, 2017; Schwager et al. 2007; Politi et al. 2012; Kuhn-Nentwig and Nentwig 2013; Wolff and Gorb 2013). There is of course highly relevant research on other spider species as well, but there is certainly not a single one species having been studied in similar depth and breadth as *C. salei* (and to a lesser degree its two allies).

### Mechanoreceptors—types of sensors

Spiders are well equipped with high-quality sensors located on and in their exoskeleton, that is at the interface to the environment. In *Cupiennius* and most likely in the majority of other spiders as well the sensory cells in the body´s periphery by far outnumber the neurons in the central nervous system. This underlines both the importance and refinement of the sensory periphery. As has been amply shown substantial pre-processing of information by the sensory receptors is a common phenomenon. It saves the central nervous system a lot of processing and integration of the sensory input (Barth 2019). Interestingly, the particular relevance of the sensory periphery in spiders such as *Cupiennius* is also underlined by the fact that the number of neurons estimated for the brains of locust and honeybee are larger by a factor of almost 4 and 10, respectively (spider: Babu and Barth 1989; Barth 2016;locust: Burrows 1996; bee: Witthöft 1967).

Although in some groups of spiders, an outstanding example being the jumping spiders, vision is a dominant sense, overall the mechanical senses are especially well developed and of corresponding behavioral significance. They respond to different types of force, like that of airflow, touch, substrate vibration and proprioreceptive input resulting from the spiders´ own locomotion (Seyfarth 1985; Barth 2002a). Some mechanosensors like the trichobothria, which respond to the slightest whiff of air (Barth 2014), and the slit sensilla, which respond to minute strains in the cuticular exoskeleton (Barth 2012b; Blickhan et al. 2021), show absolute sensitivities at the limit of the physically possible. The reader interested in details is referred to the literature, which summarizes our present knowledge of spider mechanoreceptors and highlights their technical perfection (Barth 2004, 2014; Humphrey and Barth 2008; Fratzl and Barth 2009; Barth 2012a, b). Spiders, like other arthropods, not only have mechano-sensitive cuticular *exteroreceptors* (hair-like sensilla or setae) but also *proprioreceptors* (slit sensilla, kind of embedded biological strain gages in the exoskeleton; hair-like sensilla; internal joint receptors) usually found in increased density near the joints. However, spiders do not have the equivalent of insect chordotonal organs, nor of vertebrate muscle spindles and Golgi tendon organs. In the following, some examples of the involvement of mechanosensitive spider sensilla in various motion behaviors will be given.

### Walking on solid substrate—a wealth of proprioreceptors

Spiders spread their body weight across eight legs which need to be coordinated. As has been known for a while, like many arachnids they exhibit the “alternating tetrapods” walking pattern, moving the legs in groups of four with those diagonally opposed roughly in synchrony. The most common step sequence of *Cupiennius salei* is 4–3-1–2, the numbers indicating legs 1 to 4 moving in a forward wave. Whereas leg pairs 1 and 2 pull, leg pairs 3 and 4 push (Seyfarth and Bohnenberger 1980; Seyfarth 1985; Brüssel 1987; Barth 2002b). However, the exact time at which a particular leg is moved can vary considerably, depending on walking speed, adjustment to the profile of the substrate and on the rather common loss of one or even two legs (Wilson 1967; Seyfarth and Bohnenberger 1980; Seyfarth 1985). Obviously then, sensory information and reflex control is an important aspect of spider locomotion, allowing for the continual adjustment to changing mechanical conditions, which does not result from a rigid program in the central nervous system.

The question then is which sensors play a role here, enabling the spider to adjust to a broad spectrum of conditions. There are many proprioreceptors (Seyfarth 1985; Barth 2002b) providing the spider with a detailed picture of its movements. Their feedback information to the central nervous system most likely massively contributes to the fine adjustments of movement. Such adjustment is for instance seen during prey capture, walking over rough terrain, walking on inclined and vertical surfaces or upside down, or during the spinning of an egg sac or a web. The versatility of motion also reflects the fact that spiders have at least 30 muscles per walking leg (Ruhland and Rathmeyer 1978), whereas insects and crustaceans have only about 10 and 16, respectively. It also reminds us, that spider muscles are innervated by multiterminal and many more motoneurons than the muscles of insects (Seyfarth 1985). In addition, spiders apply a sophisticated hydraulic system for the extension of important leg joints (femur/patella and tibia/metatarsus joints lack extensor muscles; see Blickhan and Barth 1985; Liu et al. 2019).

The sensor types, which supply the leg joints, are (*i*) the slit sense organs, among them, in particular, the compound or lyriform organs (Barth and Libera 1970; Barth 2012a, b; Schaber et al. 2012), (*ii*) large numbers of proprioreceptive hair-like sensilla (innervated setae) (Barth 2015, 2016; Schaber and Barth 2015) and (*iii*) a few stout cuticular bristles bridging the joints. In addition, there are (*iv*) internal receptors located close to joints. They form clusters of 3–13 multiterminal receptor cells with their dendrites ending below the joint membrane and monitoring joint position and movement (Foelix and Choms 1979; Seyfarth 1985; Seyfarth 1985). So far there is no compelling evidence for the existence of muscle receptor organs in spiders, although their occurrence still seems to be a possibility as indicated by the existence of small muscles with unusually (compared to normal leg musculature) small fiber diameters of 18–30 µm (Parry 1960; Ruhland and Rathmeyer 1978; Seyfarth 1985; Seyfarth et al. 1985). Scolopale organs such as chordotonal organs, widespread in insects and crustaceans, do not occur in spiders and other arachnids. However, as is well known from insects, there are distinctly grouped (*v*) hair plates on the coxae stimulated by the rolling over of the pleural membrane during lateral leg movements (Seyfarth et al. 1990). There are two such hair plates on the chelicerae of *Cupiennius salei* as well (Barth 2016). For anatomical details of the leg nerves and the muscles of *Cupiennius salei* walking legs and representative for spiders in general see Seyfarth (1985).

Seyfarth and Pflüger (1984) extensively studied the dorsal hinge joint between tibia and metatarsus of the walking leg of *Cupiennius salei* to describe its proprioreceptor outfit in detail. Two paired muscles provide the bending of this joint. Hemolymph pressure is used for its hydraulic extension as is also the case at the femur/patella joint, the other “knee “ of the spider leg (predominantly showing flexion and extension movements), and in the metatarsus/tarsus joint. There are four lyriform slit sense organs at the distal end of the tibia. The three lateral ones (HS8 and HS9 on the posterior aspect of the tibia and VS4 on its anterior aspect) are located in an area of the exoskeleton where they are compressed and thus stimulated when the joint is bent by the flexor muscles against a mechanical resistance as to be expected during locomotion (Bohnenberger 1981). Thanks to the PhD work by Reinhard Blickhan (Blickhan and Barth 1985; see also Blickhan et al., this issue), which enabled us to measure exoskeletal strains at the relevant locations of the lyriform organs (and other slit sensilla) in freely walking spiders, we know that the lateral organs are indeed stimulated during joint flexion, mainly during the support phase of the step. The ventral organ (VS5) is only stimulated by strains due to increased hemolymph pressure during joint extension. This implies that the presence of the four lyriform organs allows for the distinction of the phases of the stepping cycle and between strains due to muscular force and hemolymph pressure, respectively. Considering the uniqueness of the interaction between muscular contraction and hydraulic mechanisms in spiders this is a finding of particular relevance.

Surprisingly, normal locomotion and leg coordination of *Cupiennius salei* is not or only little affected by the ablation of lyriform organs on femur and tibia, nor even by cutting the major sensory nerves in tibia and femur. The basic rhythmic forward–backward motion was maintained after the operation and the untreated legs remained unaffected by it. It is difficult to demonstrate a deficit even after such drastic operations (Seyfarth and Barth 1972; Seyfarth and Bohnenberger 1980; Seyfarth 1985). One either deals with a remarkable plasticity of a central nervous program or/and a remarkable redundancy of the sensory periphery. Clearly, such general statements ask for more research. Deficits, however, can well be shown regarding proprioreceptive muscle reflexes in the walking legs (Seyfarth 1978a, b; 1985) and, more surprisingly, in regard to kinaesthetic orientation (path integration) (*Cupiennius salei*: Seyfarth and Barth 1972; Seyfarth and Bohnenberger 1980; *Pardosa amentata*: Görner and Zeppenfeld 1980). This is discussed in the following.

### Idiothetic (kinesthetic) orientation—lyriform slit sense organs involved in path integration

**Fig. 2:**
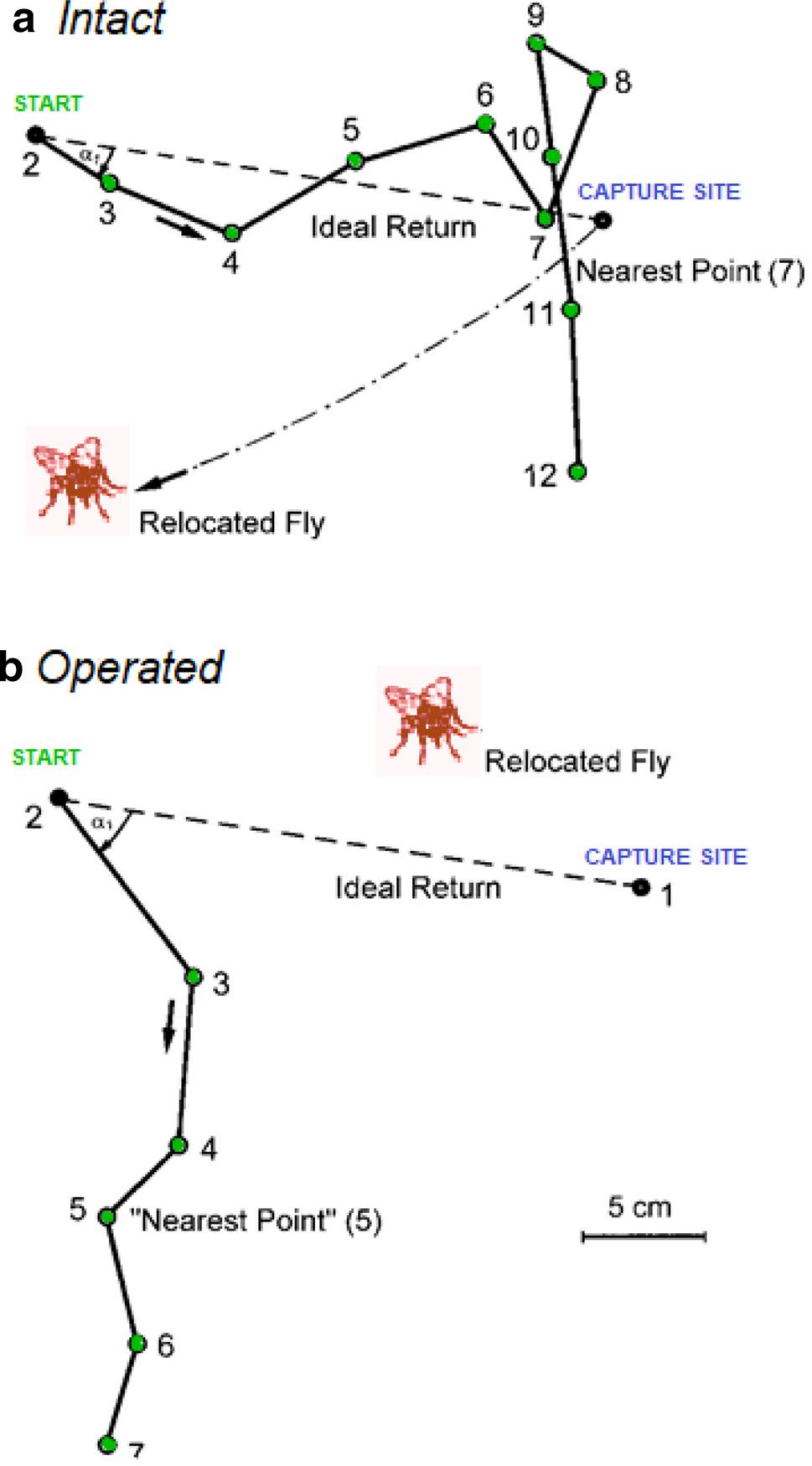
An early experiment on kinesthetic (idiothetic) orientation of *C.salei*. Example of a return path of *C. salei* from “start “ to the site of prey capture after the relocation of the fly. **a** Intact animal*,* successful return; **b** animal with all tibial lyriform organs destroyed, unsuccessful return. At point 1–12 the spider paused and/or turned. (modified: **a**, **b.** From Seyfarth and Barth 1972; with permission of SpringerNature)

Imagine an experiment bringing together a hungry spider and a stationary buzzing fly. Alerted by its trichobothria, which respond to the slightest movement of air (like that produced by the fly), the spider will run towards its prey and catch it. When separated from the fly (which can be done by delivering an electric shock through the fly) and having been gently driven away *Cupiennius salei* shows a remarkable behavior. After a few minutes´ rest and the complete removal or relocation of the fly the spider turns around and walks back to the site of prey capture (Fig. 2a). It does this in complete darkness and with its eyes covered, without the use of a dragline, chemical or tactile and gravitational cues and from a distance of at least 70 cm (Seyfarth and Barth 1972). The “homing” spider orients itself on its way back to where the fly was using stored information on its previous movement sequences, without relying on external references. This is why such behavior is referred to as idiothetic orientation (Mittelstaedt and Mittelstaedt 1973; Mittelstaedt 1985). Carefully executed ablation experiments proved the involvement of identified proprioreceptors for the first time in an arthropod: The spider’s orientation is strongly impaired after the inactivation of lyriform organs on tibia and/or femur (Seyfarth and Barth 1972; Seyfarth et al. 1982). (Fig. 2b).

To reliably judge the orientedness of the spider’s motion and to quantify it three criteria were selected: (*i*) the frequency of arrival at the correct place, (*ii*) the starting angle of the return path and (*iii*) the route taken. Organ inactivation affects all three parameters. The femoral lyriform organs have an even more pronounced effect than the tibial organs. The relevance of both is underlined by the control animals with holes in the nearby cuticle but intact lyriform organs. They show the same perfection as intact animals. According to experiments where the spiders were driven away from the site of prey capture through a semicircular corridor the way back to the goal is very close to the ideal straight route, saving about half of the distance through the corridor. This kind of detour compensation has been described for many animals, among them other spiders (Görner and Claas 1985; Mittelstaedt 1985). According to present understanding, the navigation of *Cupiennius* underlying its return to the site of prey capture is “route/path integration “, as in case of the homing behavior of other arthropods like desert ants, the champions of this art (Wehner 1992, 2020). Information about the angle and distance of the outward journey may come from different sources in the natural situation, such as visual ones like the sky compass or landmarks—or as directly shown in *Cupiennius*—be truly “kin-esthetic” information, that is information on actual movement sequences. More details are found in the original literature and a review chapter in Barth (2002b). It would certainly be worthwhile to extend these experiments to learn more about the underlying neuronal and sensory mechanisms. Attention should also be given to the integrative activity of the spider central nervous system (Görner and Claas 1985; Mittelstaedt 1985; Hartmann and Wehner 1995; Wittmann and Schwegler 1995). It has not been studied in any detail for almost half a century since the first experimental proof of the involvement of identified kinesthetic sensory organs in idiothetic orientation.

### *Muscle reflexes* in the walking legs—movement versus force receptors

The involvement of lyriform organs in the triggering of leg reflexes, assumed to be relevant for adaptive walking and leg coordination, is a rather old story, too (Seyfarth 1978a, b;1985; Seyfarth and Pflüger 1984; Barth 2002b) and unfortunately nothing really new has been added, except a few findings related to the spinnerets (see below).

In short, there are two kinds of proprioreceptive muscle reflexes: the well-known resistance reflex, which resists the imposed force, and the synergistic reflex, which is an avoidance and protective reflex withdrawing the body or its affected part from the stimulating force. By simultaneously recording the activities of both the sensilla and the leg muscles in *Cupiennius salei* and *Aphonopelma* sp. it was shown that resistance reflexes are elicited by the stimulation (imposed joint movement) of internal proprioreceptors, whereas synergic reflexes needed the stimulation of lyriform organs. Muscles were activated in the stimulated leg only by both types of reflexes (Seyfarth 1978a, b).

The *resistance reflex* did not disappear after the removal or inactivation of the external proprioreceptors, both hair-like sensilla and lyriform organs, at the affected joint (on leg segments patella, tibia, tarsus) but disappeared after sectioning the sensory leg nerve containing the axons of the internal sensory cells below the joint membrane. Resistance reflexes are well known to be involved in the fine control of locomotion (negative feedback control of limb position) in many arthropods as discussed by Seyfarth (1985) and Barth (2002b).

*Synergic reflexes* were found to activate extrinsic muscles, not however the intrinsic muscles of the stimulated joint. To give an example: Patellar muscles are activated to move the tibia away from an external force moving the metatarsus sideways against the fixed tibia. The cuticular strain due to the imposed movement and effectively stimulating lyriform organs is reduced by the reflex. Thereby the imposed movement is reinforced and the leg pulled away, which most likely has a protective function. The reflex fails after the destruction of specified lyriform organs at the tibia/metatarsus joint.

*Cupiennius salei* is literally covered by thousands of mechanosensitive hair-like sensilla (setae) (Barth 2016). Touching (deflecting) such sensilla usually leads to a withdrawal of the stimulated leg, as can be seen easily in many other spiders as well. Whereas upon tactile stimulation only the stimulated leg is visibly affected (no response of muscles in another ipsi- or contralateral leg) (Seyfarth and Pflüger 1984) body raising behavior is different. It was analyzed in detail and is shortly reviewed below in a separate section.

As pointed out and reviewed by Seyfarth (1978b) there seems to be a general tendency across the arthropods that “force receptors “ such as insect campaniform sensilla and spider slit sense organs elicit stimulus-synergic responses, whereas “movement receptors “ such as hair-like sensilla, muscle stretch receptors and chordotonal organs are dominating the resistance reflexes.

*Cupiennius* is equipped with some particularly long tactile hair-sensilla on many parts of its body. These are the outposts of its tactile sense. Their stimulation by deflection of the hairshaft elicits a variety of stereotyped movements, all needed for the spider’s natural daily life behavior and easily observed (Seyfarth and Pflüger 1984; Friedrich 1998, 2001; Barth 2016). Examples are: the raising of the opisthosoma by stimulation of such long tactile “hairs” ventrally on the opisthosoma; lowering of the body by stimulation of them just behind the eyes; withdrawal of the spinnerets and more. An interesting feature of these reflex behaviors is their stepwise nature due to an additive effect of repeated stimulation. Ventrally on metatarsus and tarsus the same type of tactile sensilla is found. Most likely they monitor the leg’s contact with the substrate and serve step control and fine adjustment during locomotion. Among all the tactile hairs tested, these are the ones most easily deflected indicating a corresponding mechanical sensitivity (Barth 2016). Neuroethological research on their involvement in locomotion control would certainly be rewarding.

### Joint movement—monitored by sensory “hairs” (setae)

**Fig. 3:**
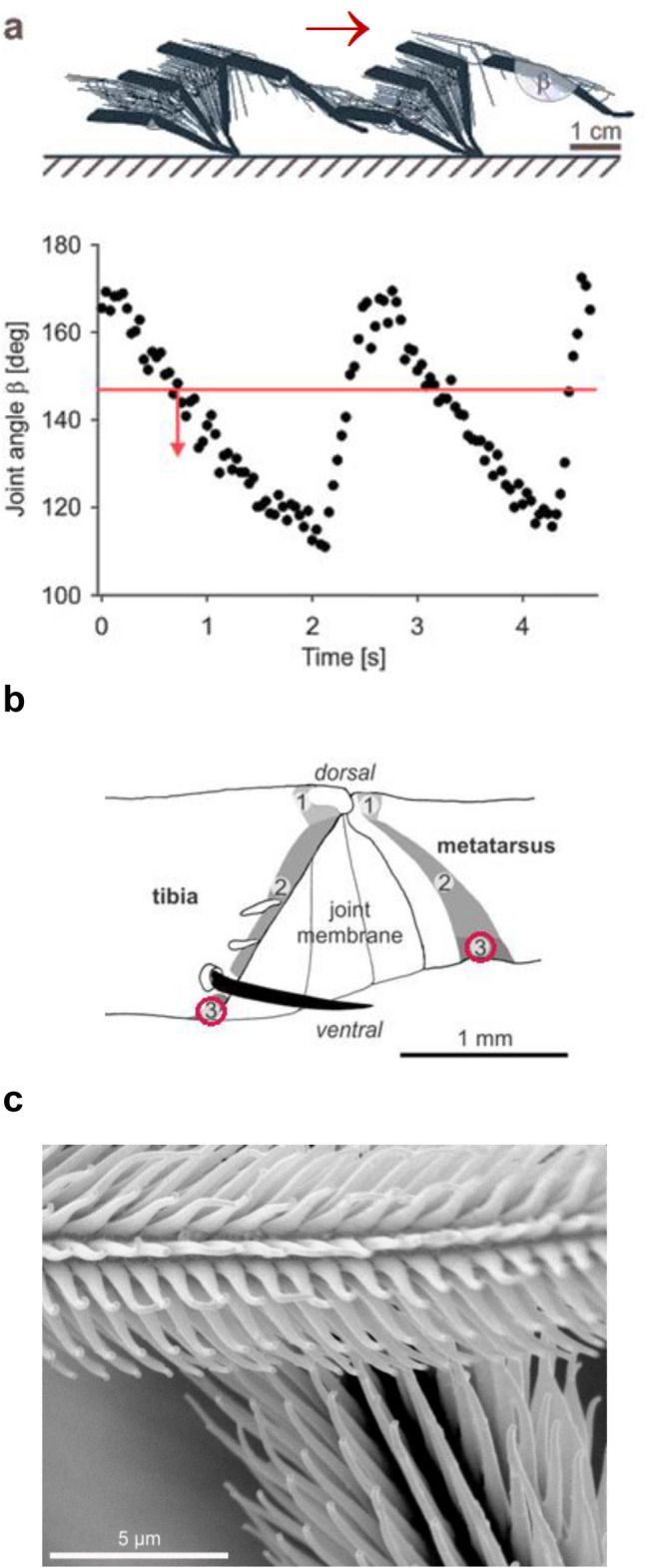
Proprioreceptive hair sensilla of *C. salei* at the tibia-metatarsus joint. **a** Movement of the joint (note joint angle β) during slow locomotion at a speed of 2 cm/s; frame-to-frame video analysis. Red line indicates the mean angle at which the hair sensilla start to deflect each other. **b** Position of the proprioreceptive hair sensilla ventro-laterally on tibia and metatarsus (see red circles). **c** SEM picture showing the microtrichs on the hair shaft of the tibial (top) and metatarsal sensilla (below) at a joint angle β of 135° (with permission of SpringerNature; modified from Schaber and Barth 2015)

Although of obvious relevance in the context of locomotion, an example of the working and proprioreceptive adaptations of sensory setae at the joints of the spider leg has been studied in some detail only recently (Schaber and Barth 2015). Ventrally on the joint between tibia and metatarsus there are hairs (some 20 on the tibia and c. 75 on the metatarsus) opposing and deflecting each other when the joint flexes. At the end of the swing phase, when the tarsus touches the substrate, the joint angle measures a maximum of c. 178°. The smallest angle (at the end of the power stroke) is only c. 124°. The mean amount of joint flexion, therefore, is about 60° during unrestrained slow forward walking. At walking speeds between 1 and 20 cm/s the stepping frequency is 0.3–3 Hz. Among the many setae found proximally and distally at the joint and deflected by joint flexion during locomotion ventral hair sensilla, which deflect each other reversibly by up to 30°, were studied in regard to their functional morphology, mechanical directionality and physiological responses to natural stimulation. Their adaptedness to proprioreceptive function clearly emerged (Fig. 3a–c).

(i) The shaft of the setae (hairs) is covered by thousands of microtrichs arranged in rows parallel to the shaft axis and in high density (15 per µm hair length in the tip region). These microtrichs (protuberances) are up to 5 µm long and c. 1 µm in diameter, with a small “hook” at their tip. While deflecting each other the opposing hairs on tibia and metatarsus are reversibly interlocked by their elaborate microtrichs, which enhance the friction between them. (ii) The structure of the hair sockets selectively facilitates large deflection in the behaviorally relevant direction. Torque measurements reveal a pronounced mechanical directionality, the setae being much more easily deflected in the direction of natural proprioreceptive stimulation than in all other directions. Torque values reached with deflections below 50° measure up to about 1 nN m in the “natural” direction, whereas in the opposite direction values of 8 nN m and more were measured, even at much smaller deflection angles. The corresponding torsional restoring constants S for both directions differ by one to two powers of ten (tibia setae: 5.92 × 10^–10^ and 3.69 × 10^–8^ Nm/rad; metatarsus setae: 8.03 × 10^–10^ and 1.51 × 10^–8^ Nm/rad). (iii) When applying roughly natural stimulation of the joint using sinusoidal half-wave deflections (resulting in a hair-shaft deflection angle of 30°) the sensory neurons supplying the setae show rapidly adapting bursts of action potentials only during the deflection phase away from their resting position. There is no response to the static deflection. These joint “hair” sensilla are typical movement receptors monitoring joint flexion at every step. Particularly at low stepping rates, the velocity of joint flexion is well reflected by the action potential rate. Thus these “joint hairs” very likely serve the local feedback fine control of the joint movement.

### Body raising—distributed neuromuscular control

**Fig. 4:**
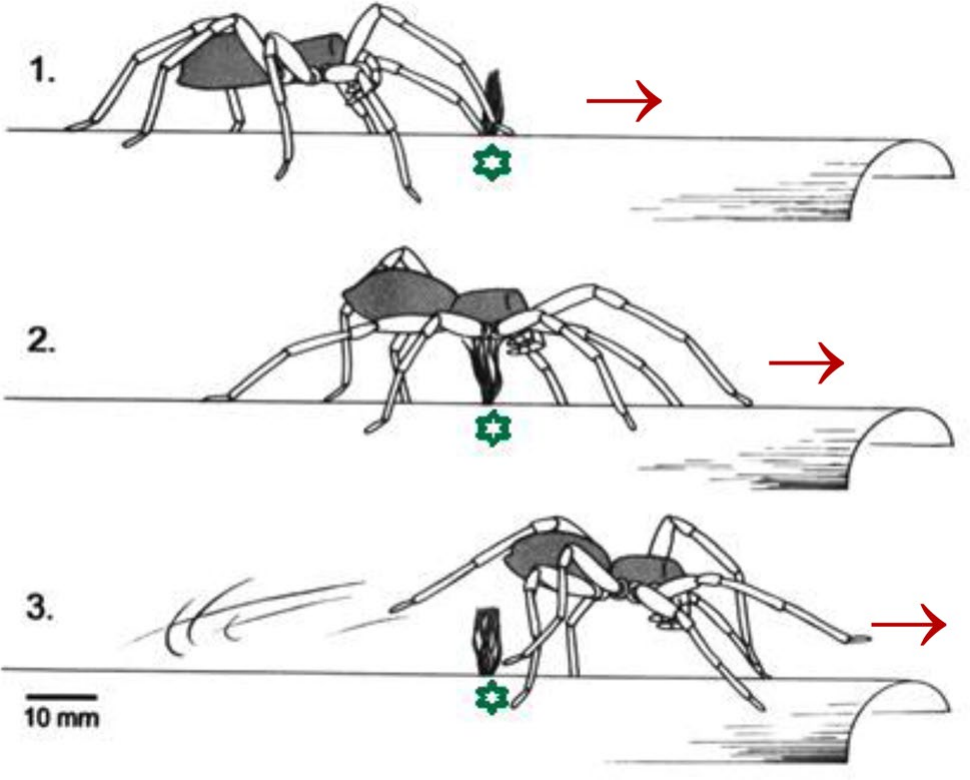
Body raising upon tactile stimulation by an obstacle. **a** The spider approaches (arrow) the obstacle (above green symbol) from the left side. **b** It raises its body upon tactile stimulation of mechanosensitive hair sensilla located ventrally on its legs and prosoma. (With permission from Aarhus University Press; modified from Seyfarth 2002)

The most intensively studied case of motion control by the sense of touch in *Cupiennius salei* (and in four other species of *Cupiennius*, in the jumping spider *Phidippus regius* and the theraphosid spider *Brachypelma* sp*.*) is the raising of its body when it meets an obstacle protruding from the ground and passes over it (Fig. 4), which is a common event (review: Seyfarth 2002). Although stereotyped and easy to elicit this behavior is not a “simple “ reflex as shown by a neuroethological analysis of the underlying flow of information, both in the sensory periphery and the central nervous system (Eckweiler and Seyfarth 1988; Milde and Seyfarth 1988; Seyfarth 2002). As opposed to local reflexes, which allow for rapid motor responses, the body raising behavior requires processing in the central nervous system and results from a distributed neuromotor control.

The sequence of events is as follows. (i) Stimulation of tactile hair sensilla ventrally on the proximal leg segments and/or sternum activates the *levator coxae* muscle of the stimulated leg. (ii) As a consequence, the coxa is pulled against the prosoma and the stimulated leg is extended hydraulically. (iii) The activity of the coxal levator muscle stimulates internal proprioreceptors at the tergo-coxal joint, which in turn triggers the simultaneous extension of the seven remaining legs. (iv) According to neuroanatomical studies (Babu and Barth 1984, 1989; Anton and Barth 1993; Ullrich 2000; Seyfarth 2002) and intracellular recordings from neurons in the subesophageal ganglionic mass the primary afferent fibers end ventrally in the suboesophageal ganglion and largely remain in the ipsilateral leg neuromer. The somata of the motorneurons activating the coxal muscles, however, are located about 200 µm away in the dorsal motor area. They are believed to be connected to the afferent endings monosynaptically, an important argument being a delay of only c. 30 ms of the local response following the tactile stimulation. There are both mono- and plurisegmental spiking interneurons extending into several leg neuromers and eliciting leg extension. Presumably they distribute the activity of the internal joint receptors to all legs. Most interestingly, there are also non-spiking pre-motor interneurons with longer-lasting graded potentials reflecting the graded character of the body raising behavior.

### Active courtship vibrations—signaling with two out of 36 muscles

Substrate vibrations are an important source of information for spiders. They lead the hungry spider to its prey and to its sexual partner and also warn of predators. When *Cupiennius* is courting on a plant like a banana “tree “ the male and the female use self-generated vibrations to communicate over distances of up to several meters to decide on the right partner and to find each other. The neuroethology of this courtship and vibratory communication has been analyzed in depth and reviewed (Barth 1997, 2002b). This includes the metatarsal lyriform organ, which is the highly sophisticated main vibration receptor showing manifold adaptations to the natural behavior of *Cupiennius* (Barth 2004, 2009, 2012).

**Fig. 5:**
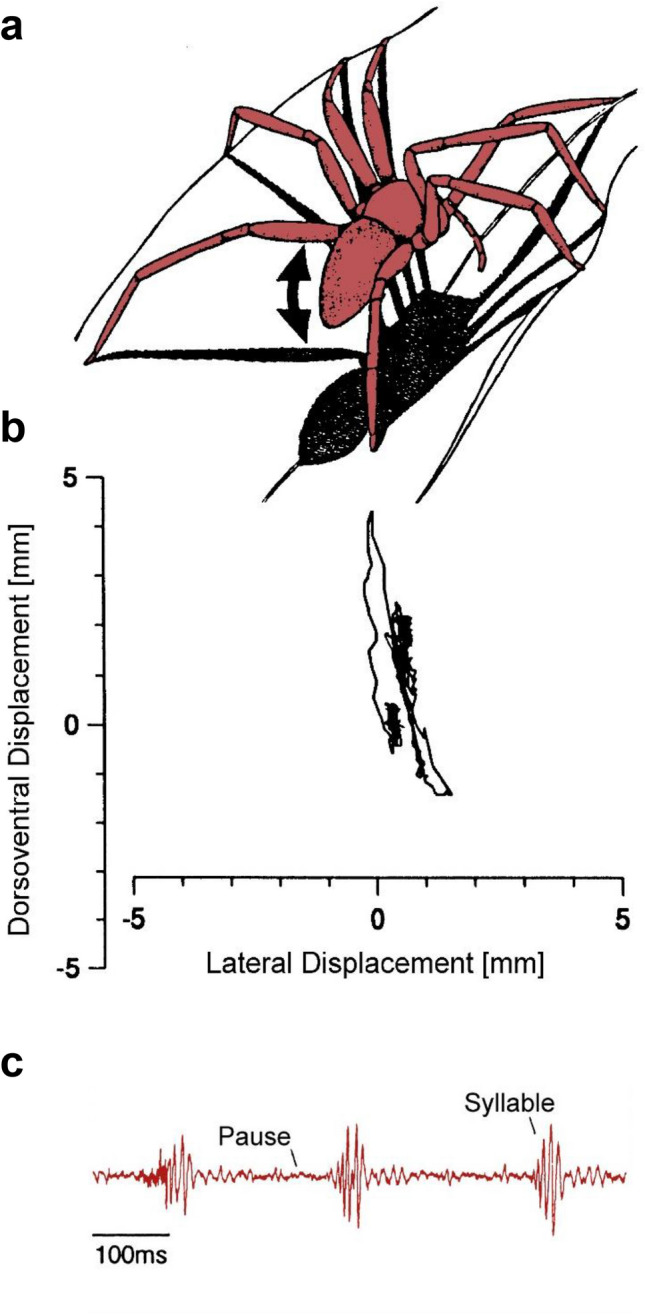
Vibratory courtship signals. **a**,** b** Movements (arrows) of the opisthosoma (*C. getazi*) of a courting male. Note that the opisthosoma does not touch the substrate. **c** Vibrations introduced by the spider into the substrate (leaf of a bromeliad). (with permission of SpringerNature (**a**, **b**) and Birkhäuser (**c**); **a**, **b.** Dierkes and Barth 1995; **c** Barth 1997)

A few aspects of the motor side shall be summarized here to explain how the spider moves to generate its vibratory courtship signals. In *Cupiennius* these signals are highly structured and critical for species identification. They come in syllables similar to cricket song and are produced by an up and down movement of the opisthosoma without touching the substrate (Fig. 5a–c). The opisthosomal vibrations are introduced into the substrate (the plant) through the legs. The carrier frequency in the male signal is 80–100 Hz and the female’s innate releasing mechanism is tuned to a specific combination of syllable duration and the duration of the pause between the syllables (Schüch and Barth 1990; Baurecht and Barth 1992). Regarding the opisthosomal motion there are several questions. What does the exact movement look like? Which muscles are involved? Is the main frequency of the male signal of 80–100 Hz a direct result of muscle contractions at this frequency or are passive mechanisms like resonance involved? The answers are as follows (Dierkes and Barth 1995).

Using high-speed video analysis and laser Doppler vibrometry the freely courting males were shown to move their opisthosoma about an axis located in the petiolus and almost perfectly up and down with only little lateral movement. Taking the spinnerets as a reference the amplitude is c. 2° (or c. 0.4 mm) at the beginning of a series of syllables. It increases to c. 30° (c. 6 mm) in the three to four final syllables of the series, without the opisthosoma ever touching the substrate. According to spectral analysis the movement contains a low-frequency component of 10–20 Hz and a superimposed component of c. 80 Hz. The latter component occurs during the upswing of the opisthosoma and is closely correlated with the appearance of the corresponding substrate vibrations. There are no resonances in a typical dwelling plant in the relevant frequency range. Transfer functions of various body parts of the spider, beginning with the leg tarsi (which pick up the substrate vibrations), show resonances between 0 and about 250 Hz (Dierkes and Barth 1995). Such measurements are hard to do with the spider under sufficiently natural conditions and assuming a controlled body posture. The significance of these body resonances should, therefore, not be overestimated yet.

Electrophysiological recordings of the muscle activity in courting males clarified the origin of the high-frequency component of the male courtship vibration. There are as many as 36 muscles in the petiolus region potentially involved in the opisthosomal courtship movements (Dierkes and Barth 1995). According to the specifics of its attachment, only muscle 81 looks like a levator muscle. Muscle 85 (a large paired muscle) is the depressor. It was found to be active during courtship only, but not during locomotion. According to the electrophysiological recording from muscle 85 and the simultaneous monitoring of both the opisthosoma movement and the substrate vibrations the 80 Hz component of the courtship vibrations is not due to plant resonances but actively produced by the spider. A downward movement of the opisthosoma is initiated by a first action potential of the depressor muscle 85. Its subsequent discharges go along with a tetanic muscle contraction which keeps the opisthosoma in its ventral position. While the levator muscle (most likely Nr.81) moves the opisthosoma upward again depressor muscle 85 contracts three to five times: It thereby moves the opisthosoma slightly downward three to five times at high velocity. This is how the main frequency component of 80–90 Hz of the courtship signal is generated. The high contraction frequency of muscle 85 is in the range of the resonances of the spider body. The vibratory signal may thus be amplified on its way through the body into the plant (see above), but we are still largely ignorant regarding the effect of leg positions, muscle tensions, and hemolymph pressure on details of vibration transmission through the spider body.

How about the sensory side of this motion? The male starts generating vibratory courtship signals when it comes across a female dragline. It probes the dragline with its pedipalpal chemoreceptors and perceives the female sexual pheromone (S-dimethyl ester of citric acid) attached to it (Gingl 1998; Schulz et al. 2000; Tichy et al. 2001; Barth 2002b). Whether the prominent slit sensilla found on the petiolus or/and the small slit sensilla found ventrally on the opisthosoma (Libera and Barth 1970; Barth 1985b) play a role in the fine tuning of the opisthosoma’s motion is still an open question.

### Jump into the air—airflow guides to flying prey

**Fig. 6:**
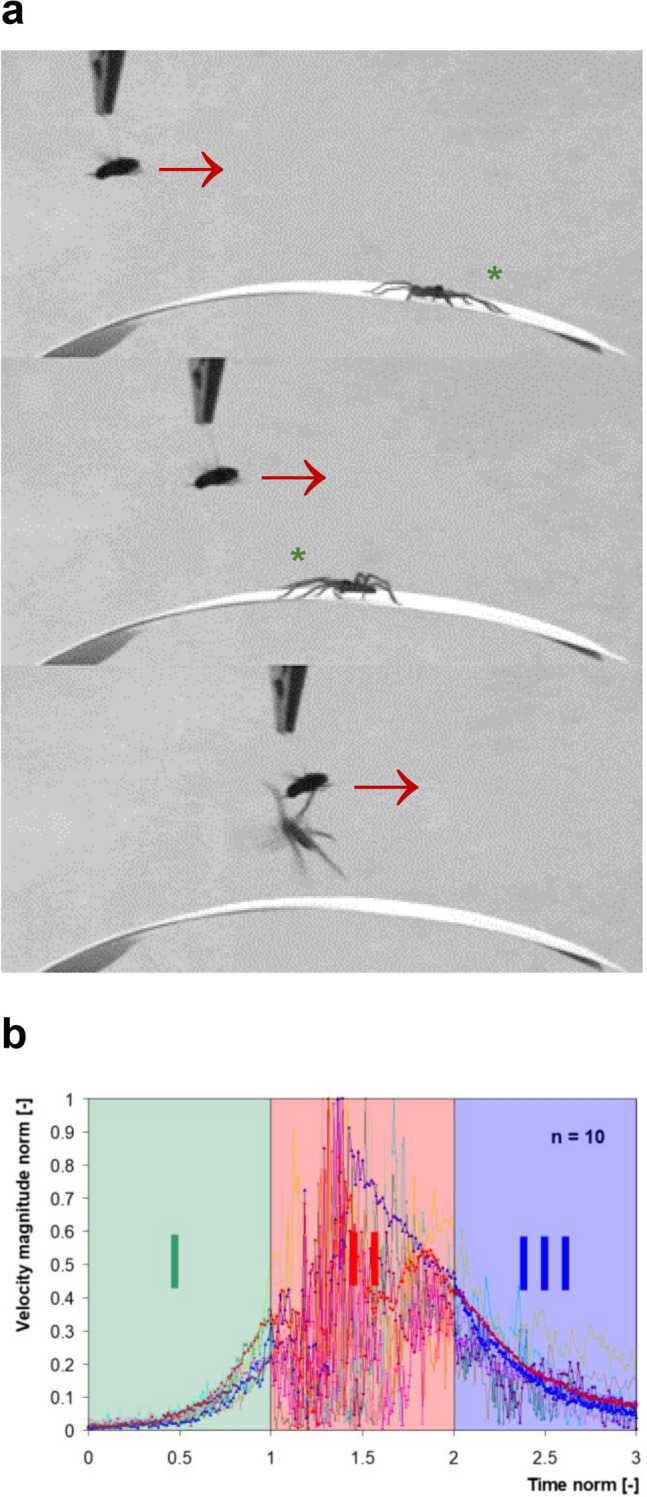
Catching flying prey. **a** A tethered humming fly approaches (arrow) a juvenile *C. salei* sitting on a bromeliad leaf with its front (green asterix) oriented away from the fly (above). The spider turns towards the approaching fly when it is still a few centimeters away (middle). It finally jumps into the air to catch the fly when its position is above the closest tarsus (below). **b** Airflow measured above the spider tarsus and showing the abrupt change in flow turbulence which triggers the spider’s timely jump. (with permission of SpringerNature; modified from **a** Barth 2014; **b** Klopsch et al. 2013)

*Cupiennius salei* and its two big relatives (*C. getazi, C. coccineus*) are “sit and wait- hunters”. Being alerted by vibrations of the substrate (a plant) they commonly wait motionlessly until the prey (such as a cockroach or an earwig) producing them comes within the reach of a jump. The spiders change from being motionless to moving like a flash within milliseconds (Melchers 1967). Maybe the most spectacular kind of prey capture motion of *Cupiennius* is when it catches flying prey from the air (Fig. 6a, b).

One of the several questions associated with this motion behavior is: Which cues and information uses the spider when jumping into the air, well oriented and at just the right moment in time to catch a freely flying fly passing by at a speed of 1 m/s and more? The most relevant and fully sufficient cue comes from the airflow generated by the flying prey. The natural stimulus is multimodal but by careful experimentation the necessity of visual input, substrate vibration and airborne acoustic stimulation could be safely excluded and the dominance and effect of airflow alone confirmed. Being deprived of its airflow sensors, *Cupiennius* never jumps into the air to catch prey, but it readily does it with its eyes covered. The functional morphology, physiology and biomechanics of the airflow sensors, the trichobothria, have been intensively studied (reviews: Humphrey and Barth 2008; Barth 2014). Trichobothria are the functional equivalent of the analog insect “filiform hairs “. They are extremely sensitive. As seen by the action potentials generated by their sensory cells, they are deflected and stimulated by frictional forces (at a threshold deflection of the hairshaft by 0.01° and forces in the order of 0.4 to 4 × 10^–6^ N) resulting from the slightest movement of air (velocities as slow as 0.15 mm/s). The elastic restoring force at the articulation of the hair shaft is extremely small as is the corresponding spring stiffness (in the order of 10^–12^ Nm/rad).

*Cupiennius* has some 100 trichobothria dorsally on the tibia, metatarsus and tarsus of each leg. Including the pedipalps this comes to a total of close to 1000 trichobothria, which is the largest number so far known of any spider. In general, wandering spiders have more trichobothria than web spiders for which the airflow-sense seems to be much less important behaviorally. We have never succeeded in eliciting a similar jump in any web spider by an airflow stimulus (Barth 2014, p. 173).

Technology like high-speed video analysis and digital particle image velocimetry (DPIV) had to be used to find out what the relevant stimulus parameters of the airflow are, which make the spider jump (Klopsch et al.^2012^, ^2013^; review Barth 2014). An attractive prey like a freely flying fly *(Calliphora erythrocephala)* generates both a circulating airflow in front of itself and a wake pointing obliquely downward behind itself. An approaching fly first exposes the spider to its “front flow “. The velocity of this flow increases nearly exponentially with time and decreasing distance and shows very little fluctuation. Its maximum velocity at the site of the nearest tarsal trichobothria is c. 0.16 m/s. The spider reacts to this flow when the fly is still about 4 cm away by turning around towards the flow (Fig. 6a). It always turns into the direction given by the leg stimulated first but never jumps in this situation. The actual jump is triggered by an abrupt change in flow characteristics which occurs when the fly is above the tarsus (Fig. 6 b). Trichobothria are now stimulated by the fly’s wake. Different from the “front flow “ this flow is highly fluctuating and has an increased vertical velocity component. Its power spectrum indicates a shift of the main frequency component towards higher values (from c. 8–c. 18 Hz) and now contains frequencies up to 250 Hz and more, which are not usually contained in the natural background flow. The trichobothria form a strictly phasic sensory system. They respond to stimulus dynamics as opposed to static stimulation as was also found in corresponding central nervous neurons. Thus the trichobothria are sensors perfectly adapted to being activated by prey-generated highly fluctuating airflow.

As one would expect, the high-frequency components in the fly wake quickly disappear with distance. At a fly distance of ca. 25 cm the airflow is very similar to background flow and the spiders don’t jump anymore, which of course makes sense.

Considering the main focus of this Special Issue of JCP-A it may be worth noting that *Cupiennius* adjusts its leg and body position while already in the air, another remarkable motion phenomenon deserving further study.

### Dispersal by wind—initiated by trichobothria

Two types of motion still shall be mentioned. One is a “pre-ballooning “ kind of dispersal behavior, the other is rowing on the water surface. Both activities are not a daily practice of *Cupiennius* but still they are important in the relevant ecological contexts (see also Bonte 2013).

Unlike many insects, spiders do not normally fly. The notable exception is “ballooning” which may take spiderlings passively away to great heights and sometimes distances of hundreds of kilometers. As far as we know *Cupiennius* (the three large species studied) is not even “ballooning”. However, it can be readily watched to show a behavior roughly similar to what in many of its passively flying relatives is referred to as pre-ballooning (Decae 1987).

**Fig. 7:**
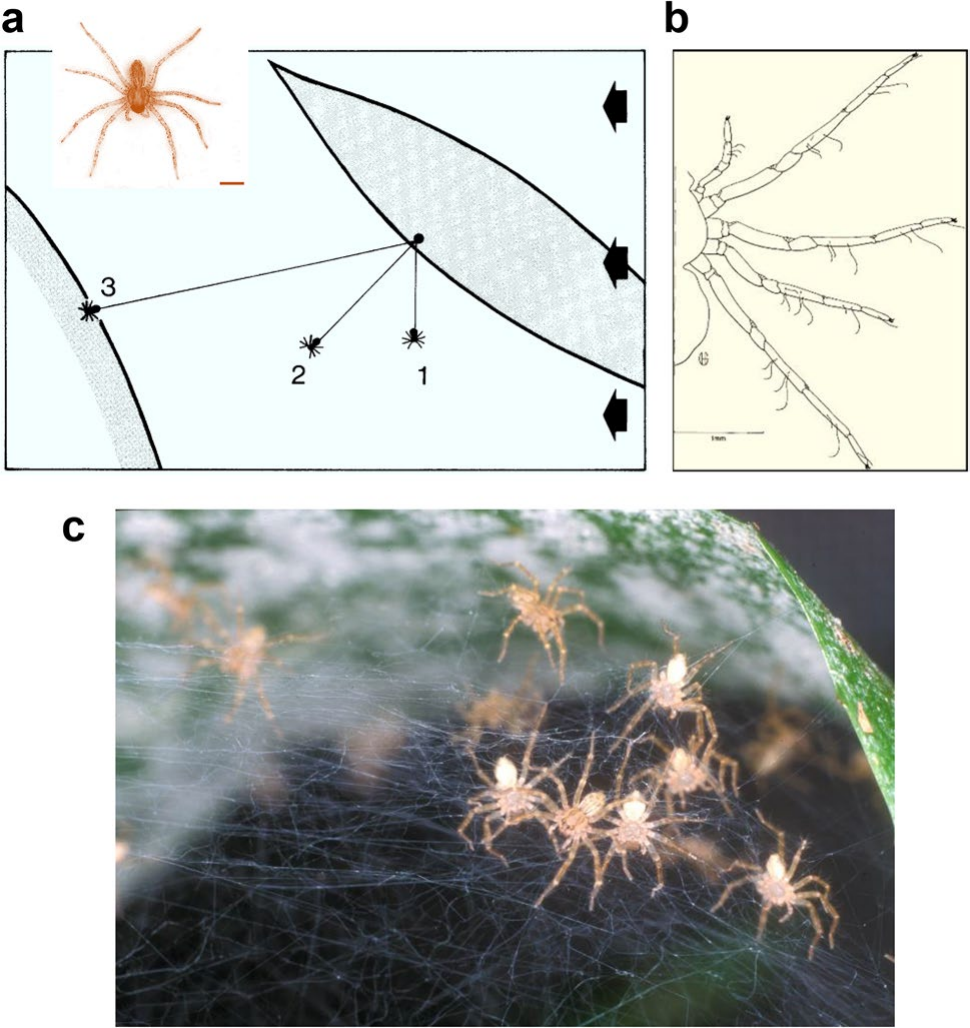
Drop and swing dispersal behavior. **a** Upon exposure to adequate airflow (arrows) spiderlings drop from their dwelling plant and swing on a lengthening thread in the wind (1,2,3). When touching a nearby substrate they attach to it. Inset: *C. getazi* spiderling, 9 days after leaving the egg sac. **b** Trichobothria of a spiderling (*C.salei*) at the age of 9 days after leaving the egg sac. **c** Spiderlings in a tangle of silken threads spun by the female at the time of the spiderlings leaving the egg sac. Scale bars in **a** and **b** 1 mm (with permission of SpringerNature; modified from **a** Barth et al. 1991; **b** Barth 2002b; **c** foto FG Barth)

About 9 days after leaving the egg sac the spiderlings of the large species of *Cupiennius* are still small, with a body length of about 2 mm and an average mass of about 1.26 mg (Fig. 7a–c) (Barth et al. 1991). By then the yolk they have been provided with is almost used up. They need food from outside now and have to deal with severe competition for resources among hundreds (up to more than 1000) of fellow spiderlings crawling around in a small tangle of threads spun by their mother around the eggsac they have left. Overpopulation has to be avoided and there is even the risk of cannibalism. What to do? The answer is the so-called “drop and swing behavior”.

Instead of assuming the tiptoe position in an elevated location as it is seen in truly ballooning spiders before take-off *Cupiennius* drops from its dwelling plant hanging on its dragline and swings in the wind with the dragline lengthening progressively. When it touches solid substrate it frequently repeats this process. The distances covered are usually small, with the spiderling landing on another leaf of the same or on a nearby plant. Therefore, the drop and swing behavior of *Cupiennius* provides a small scale dispersal only, an ecological consequence being that the spiderlings and thus the population is kept in a safe and reasonably stable habitat (Barth et al. 1991; Barth 2002b).

But what triggers the actual dropping motion of the spiderlings? The answer to this question is once more closely related to the trichobothria. *Cupiennius* spiderlings at the young age considered here have only six to seven trichobothria on each leg (as opposed to almost 100 in the adult stage) (Fig. 7b). Nevertheless, due to the fragility of the spiderlings it is hardly possible to remove all trichobothria without uncontrolled damage to the animals. However, there is good indirect evidence of the role played by the trichobothria in the drop and swing behavior. It comes from the physical conditions in the environment, which make the behavior possible and elicit it. The spiderlings were exposed to controlled airflows in a wind tunnel (Barth et al. 1991). At an age of about 9 days up to 70% of the spiderlings started to run around and to show drop and swing behavior when exposed to wind. The percentage much depended on wind speed and on the degree of turbulence of the airflow, which the spiderlings obviously are able to sense. The effective wind speed may be as low as 0.2 m/s, but at 1.5 m/s and more the spiders do not drop from the plant anymore. On the contrary, those having dropped already return to their starting point. Apart from a certain range of wind speed the other critical parameter is the degree of turbulence. The larger it is the higher is the number of responses. Thus at a mean velocity of 0.7 m/s of a quasi laminar airflow (5% turbulence only) there were only about half as many reactions than at 35% turbulence. At the same time, the most effective average response frequency shifted from 0.7 to 0.9 m/s wind speed. The wind speeds found to be effective for *Cupiennius getazi* are similar to those given for truly ballooning spiders (Richter 1970, 1971; Vugts and van Wingerden 1976; Bell et al. 2005).

Being a phasic system of receptors, only responding to stimulus dynamics, the trichobothria are particularly well suited to respond to turbulent airflow and to tell the spider when the conditions are right for dispersal. Their role in finding out about the right wind conditions is also suggested by the behavior of truly ballooning spiders which may even raise their front legs to probe the airflow conditions actively before assuming the typical tiptoe position and finally releasing their silken ballooning fibers (Cho et al.^2018^).

### A turn towards the stimulus—sensors on eight legs

**Fig. 8:**
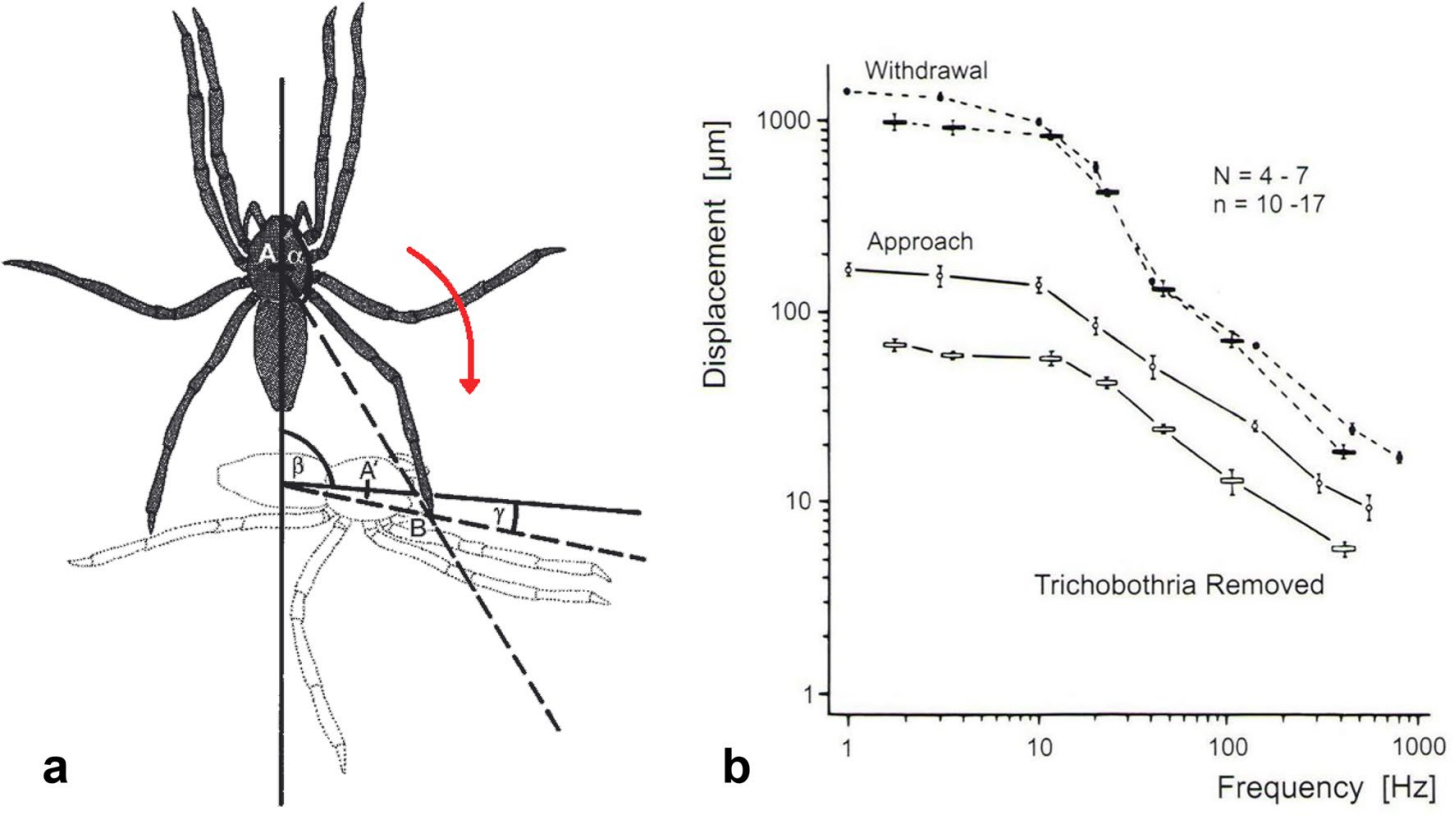
Turning towards or away from a stimulus source. **a** Turning movement (arrow) of *C. salei* towards a vibratory stimulus applied to the tarsus of its right hindleg. Quantification of the spider’s motion by measuring the parameters indicated. *α* stimulus angle, *B* site of stimulation, β turning angle, *γ* error angle, *A* center of prosoma. **b** Behavioral thresholds of *C. salei* for its approach (prey capture) and withdrawal (escape) reaction to stimulation with substrate vibrations. The approach reaction has a considerably lower threshold than the escape reaction and is lower for band-limited noise stimulation (bars) than for sinusoidal stimulation (circles). (With permission of SpringerNature; from Hergenröder and Barth 1983a, b)

Having identified a vibratory stimulus as being generated by close-by prey *Cupiennius* has to turn rapidly and precisely towards it for a successful catch. As is easily observed the spider ‘s vibratory “view” is a horizontally all-around view, making use of the radial arrangement of its eight legs, which it keeps in a stereotyped position when ready for prey capture. But how does the spider decide on the correct turning angle towards the source of vibration? In numerous experiments, different combinations of the legs were exposed to electronically controlled prey-like substrate vibrations and their effect on the turning angle studied (Hergenröder and Barth 1983b; Barth 2002a, b) (Fig. 8a). The turn towards the stimulus source is slightly more precise when it comes from in front than from behind (smaller error angle when stimulating forelegs than with stimulation of the other legs). And surprisingly, the spider turns faster for stimuli from behind than from in front, which makes sense biologically: It is crucial to reach the prey as quickly as possible. The main conclusion from many of these experiments is that the spider turns correctly on the basis of the stimulated leg combinations alone, even when they are stimulated simultaneously by identical stimuli (including no difference in magnitude). This led to the elaboration of a diagram describing the interaction of the sensory input from the legs in the central nervous system. The connectivity diagram does indeed allow the correct quantitative prediction of the turning angles of the freely behaving spider. Its important features are (i) an error angle smaller for stimulation of the forelegs than of the hindlegs, (ii) an additive ipsilateral inhibition (unidirectionally directed from front to back) between the legs of one bodyside, and (iii) a connection of the legs on opposite sides by a multiplicative contralateral inhibition (Hergenröder and Barth 1983b; Barth 2002b). A qualitatively determined model of central connectivity in another arachnid, the scorpion *Paruroctonu*s *mesaensis,* postulates ipsi- and contralateral inhibition as well, but according to present knowledge the differential weighting of the input from different legs as well as the unidirectionality of the inhibition are special for *Cupiennius* (Brownell and Farley 1979; Brownell and van Hemmen 2001).

While in the laboratory the spider turns correctly on the basis of the sensory connectivity of its legs alone, differences in magnitude and time of arrival of the stimuli reaching its different legs are part of natural stimulation, when the spider is sitting on a plant with all eight legs exposed to substrate vibrations. Again, experiments with electronically controlled vibrations allowed for a quantification of the effect of differences in time of arrival (Δt) and stimulus amplitude (Δd). *Cupiennius* consistently turns towards the leg stimulated first or more strongly as if it had been the only leg stimulated. Values of 4 ms for Δt and 10 dB for Δd had a strong effect. There are interneurons in the subesophageal ganglia which nicely reflect the whole spider’s behavioral responses (Speck-Hergenröder and Barth 1987). Because of the dispersive propagation of vibrations in a plant Δt depends on frequency. The values for exposure to female or male courtship vibrations (main frequency components 30 and 80 Hz, respectively) are still in the behaviorally effective range. For prey vibrations which contain higher frequencies the values are below 1 ms and do not elicit responses in the behavioral experiment. The scorpion *Paruroctonus mesaensis* still does react at values down to 0.3 ms (Brownell and Farley 1979). As to the Δd values: Considering the attenuation (damping) of signals propagating on typical *Cupiennius* plants the values found to be effective in the laboratory experiments are to be expected in the natural situation as well. However, due to the heterogeneity of vibration propagation in the plants the spider has to deal with a frequency-dependent spatial pattern of stimulus intensities and we do not know how the spider handles this seemingly big problem (Wirth 1984). More research is encouraged!

### Escape or attack—the interaction of two sensory systems

In *Cupiennius* prey capture behavior is easily released by substrate vibrations alone. Signals carried by airflow as well as vision are not needed. However, prey capture can also be elicited by adequate airflow signals such as those generated by a flying insect (Klopsch et al. 2012, 2013). The interaction of the two sensory systems involved, the slit sensilla and the trichobothria, is complex even though one sensory channel alone is fully sufficient to trigger/control a specific behavior. How, in the given case, does stimulation from above (airflow) interact with stimulation from below (substrate vibration)? The answers turned out to be quite intriguing (Hergenröder and Barth 1983a).

Actually, substrate vibrations elicit two opposing behaviors, depending on their magnitude and frequency. Whereas upon weak stimulation the spider shows approach/attack behavior, it withdraws/escapes following strong stimulation. The respective threshold curves (necessary displacement versus stimulus frequency) show this clearly (Fig. 8b). Surprisingly, after the removal of the trichobothria thresholds for the turning-away response (escape) rose by ca. 10 dB. Obviously, trichobothria are co-stimulated when only vibrating the substrate. Although the thresholds for the turning-towards response (attack) did not change, the response probability did. Removal of the trichobothria goes along with a decrease of the number (frequency) of negative responses, which explains itself by the corresponding shift of the behavioral threshold curve. The close relationship in particular between the negative (escape) response and the trichobothria is further underlined by the increased response time after inactivation of the trichobothria. The interaction between the two sensory systems is both behaviorally significant and complex. A simplified diagram of the interactions mainly describing current behavioral knowledge may serve as a start for future research (Hergenröder and Barth 1983a).

### Rowing on the water surface—water depth, surface tension, viscosity

Fishing spiders (Pisauridae) such as the North American *Dolomedes triton* and the European *D. fimbriatus* are comfortable on the water surface, where they hunt for prey, escape from predators and search for mates (Shultz 1987; Bleckmann 1994; Bleckmann et al. 1994). As has been well studied and reviewed they propel themselves forward on the water surface using two different gaits, rowing and a spectacular galloping. The biomechanics of horizontal thrust generation for these types of locomotion has been analyzed by Suter and Wildman (1999) and Suter et al. (1997, 2003). Horizontal thrust is mainly due to the drag resistance generated when a leg and its associated water dimple at its tip are moved over the water surface during the stance phase of the stepping cycle. Both surface tension and bow waves were found to be less important in providing the resistance to the moving legs, which is needed for locomotion on the water surface (Suter et al. 1997).

*Cupiennius*, recently put into the family of Trechaleidae, is not commonly seen on the waters of ponds or rivers. It is predominantly terrestrial, but nevertheless capable to move forward on the water surface like many of its fellow trechaleids, which—tale-tellingly—are also called fisher spiders. In addition to rowing, *Cupiennius* is occasionally seen to jump forward on the water surface. Its ability to stay and move on the water surface is believed to be advantageous during the rainy season in its neo-tropical habitats. When on the water surface and changing its normal locomotory gait to rowing, *Cupiennius* lies flat on the water with its legs extended and the surface tension bearing its weight. Its hind legs are not used for rowing and both legs of pairs 1,2, and 3 are moved simultaneously each and the pairs in succession 1–2–3 (Barnes and Barth 1991).

The question of particular interest in the present context is: Which features of the substrate and correlated sensory stimuli induce the change of gait? The answer potentially also provides a hint as to the sensory receptors involved. To find out about the switch inducing the behavioral change, the European *Dolomedes fimbriatus* (Pisauridae), a semiaquatic spider routinely moving on both solid substrate and the water surface, was compared to *Cupiennius salei*, a terrestrial wandering spider typically found on solid substrate rather than on the water. The parameters experimentally modified were water depth, kinematic viscosity of the fluid (sugar solutions) and surface tension (olive oil, water, mercury). The outcome of these experiments was as follows (Barnes and Barth 1991). (i) *Water depth*: The switch of gait can be induced not only on water but also on mercury. In addition, both species can walk in shallow water. This implies that water as such is not a condition for changing the gait. Overall, the change can be more easily elicited in *Dolomedes* than in *Cupiennius*, which reflects the difference in lifestyle. In *Cupiennius,* in particular, the water depth at which rowing can be elicited correlates negatively with the spider’s leg length (age) but not with its mass. Whereas *Cupiennius* waded at water depths as large as 10 mm and seemed to avoid rowing as long as possible (until losing contact with a firm substrate), *Dolomedes* started to row at depths below 1 mm already. (ii) *Viscosity*: The resistance during the power stroke, when the leg is retracted, depends on the kinematic viscosity of the liquid, and indeed seems to be measured by the spiders. Using sucrose solutions of different concentrations, the percentage of walking as opposed to rowing varied accordingly. It much increased at 216 cS and beyond, both in *Dolomedes* and young *Cupiennius* of the same size. *Dolomedes* started to row at lower values of viscosity and adult *Cupiennius* showed a much higher percentage of rowing and a lower percentage of walking at 216 cS than its young conspecifics. (iii) *Surface tension*: Olive oil with its very low surface tension (32 mN/m) does not sufficiently support the spiders. They simply sink into it. On the water (73 mN/m) they row, whereas on mercury with its high surface tension (435 mN/m) but lower viscosity than water *Cupiennius* mostly walks and *Dolomedes* mostly rows, but also jumps and walks. For *Cupiennius* a high surface tension affects the gait in the same way as does the contact with the substrate, that is it inhibits rowing.

Although the data at hand are providing first insights only it is tempting to conclude that slit sensilla measuring cuticular strains (Barth 2012a, b; Schaber et al. 2012) are substantially involved in inducing the change of gait. Direct evidence and the identification of the receptor location still have to be provided. Slit sensilla may also contribute information about the contact of the leg with a solid substrate. Such contacts imply a reaction force and thus strains in the exoskeleton. However, there are also highly sensitive tactile hair sensilla on the ventral surface of the tarsi, which might well do the job, alone or in addition to the slit sensilla (Barth 2014). More research on the neuroethology of the change of gait is both needed and promising.

### Outlook

As amply demonstrated by several contributions to the present Special Issue on arachnid locomotion and kinematics a number of rather technical and physical aspects have already received considerable attention. Examples are leg coordination in rhythmic walking and running, the hydraulics of leg extension at the leg’s two dorsal hinge joints (where extensor muscles cannot be implemented), ground reaction forces, strains in the exoskeleton, models of 8-legged locomotion, energy efficiency, micromechanics of adhesion, and more recently also biomimetics of spider locomotion and its application to robotics (Wang et al. 2011;Spagna and Alleyne, this issue). The interest in the sensory control of spider motions has been much more limited. This may surprise since the usefulness of all these motions critically depends on the spider’s ability to control them.

The few examples given in the present review point to the richness of non-locomotor movements and stress their relevance for the spider’s everyday life and survival. They also underline the richness and subtlety of the spider’s relation to its environment and the fundamental role of its sensory systems in controlling and adapting its different types of motion. Movements not mentioned here include those seen during copulation, the anchoring of the dragline (see Wolff 2021), the handling of prey, the spinning of the elaborate egg sac (Melchers 1963) and of the tangle of threads for the spiderlings when leaving it, movements in the narrow retreat on their dwelling plant, and more. Obviously, *Cupiennius* and other spiders are well equipped with sensors, in particular mechanoreceptors responding to different kinds of force (exerted by air flow, strain, touch, substrate vibrations, joint movements), and enabling them to manage all of these motions in an adaptive way.

To understand the behavioral significance of the spider’s myriads of sensory receptors and their synergistic and integrative interaction is both a rewarding and a challenging task. Evidently, its sensors provide the spider with a very detailed “picture” of its motions, highly resolved both in time and space. Among the challenges research faces are (i) the sheer number of the receptors, redundancies, and receptor functions in only hard to detect subtleties of behavior. An example illustrating this is the puzzling persistence of leg coordination and of the basic rhythmic motion after substantial deafferentiation by cutting leg nerves (Seyfarth and Barth 1972; Seyfarth and Bohnenberger 1980; Seyfarth 1985). Another challenge resides in the (ii) general problem with ablation experiments. Not seeing a deficit or any behavioral change just tells us that the receptors under study are not a necessary condition under the given circumstances for the behavioral performance studied. One simply might not have chosen the right behavior to detect the deficit. The ablation of lyriform organs and the following absence of an obvious effect on normal walking but the pronounced effect on kinesthetic orientation (as described above) is a telling example. A further challenge are (iii) dependencies on the circadian rhythm (Seyfarth 1978a, b; Schmitt et al.^1990^), as shown by the decreased willingness of night-active *Cupiennius* to exhibit courtship behavior or even “simple” local reflexes during daytime as compared to dusk and night time (Seyfarth 1978a, b; Barth 1997). Clearly, an adequate combination of reductionist laboratory work and general biology is strongly needed, including careful observations in the field. In spiders, a particular additional complication is a pronounced (iv) efferent control of the sensory periphery, which is well established anatomically. All the receptors addressed in the present review show profuse efferent innervation with numerous synapses on the somata and their dendrites and axons (Foelix and Choms 1979; Fabian-Fine et al. 2000, 2002) indicating neuronal integration in the sensory periphery. Octopamine and GABA_B_ were shown to enhance and to inhibit, respectively. There is evidence for other transmitters as well, acting locally as opposed by circulating as a neurohormone (Panek et al. 2003; Barth 2004; Widmer et al. 2005; Tarr et al. 2018). Unfortunately, the functional role of the efferent signal modulation in actual spider behavior still is essentially unknown. Finally, (v) multimodality has to be kept in mind. For good reasons there is a strong tendency of experimental research to consider a system in a reduced but manageable way. As a consequence, the most prominent behaviors and the most clearly involved sensory systems receive most of our attention, although several sensory systems may be involved, even though in a subtle way. Fragmenting the organism in a reductionist way to understand basic sensory and neuroethological mechanisms has been a very successful approach as is shown by a multitude of examples from all sorts of sensory modalities. However, one has to be aware, that at least in many if not all natural behaviors the spider will make use of a combination of all sensory data available.

Clearly then, the examples of sensory guidance given in the present review are just a modest beginning. There is still a long way to finally understand the interaction of all the components making up the entire sensori-motor system, which underlies a particular spider motion and makes its adjustment to changing conditions possible. We are still far from a full appreciation of the complex motor programs and of the integration and processing of sensory information in the central nervous system, needed in addition to the remarkably competent pre-processing in the sensory periphery (Barth 2019).

However, despite all this the beginnings have told us a lot already and are promising. They may well serve as a springboard for further research, keeping the organismic “whole-system” picture of natural behavior in mind.
